# Periaortic Adipose Tissue and Aortic Dimensions in the Framingham Heart Study

**DOI:** 10.1161/JAHA.112.000885

**Published:** 2012-12-19

**Authors:** George Thanassoulis, Joseph M. Massaro, Erin Corsini, Ian Rogers, Christopher L Schlett, James B. Meigs, Udo Hoffmann, Christopher J. O'Donnell, Caroline S Fox

**Affiliations:** NIH/NHLBI Framingham Heart Study, Framingham, MA (G.T., C.J.O., C.S.F.); Department of Mathematics and Statistics, Boston University, Boston, MA (J.M.M.); Cardiac MR PET CT Program, Massachusetts General Hospital, Boston, MA (E.C., I.R., C.L.S., U.H.); General Medicine Division, Massachusetts General Hospital, Boston, MA (J.B.M.); NIH/NHLBI Intramural Research, Bethesda, MD (C.J.O., C.S.F.)

**Keywords:** adipose tissue, aneurysm, aorta, peripheral vascular disease

## Abstract

**Background:**

Periaortic fat, because of its contiguity with the aorta, may promote vascular remodeling and aortic dilatation. However, the relations between perioartic fat depots and aortic dimensions have not been previously described.

**Methods and Results:**

A total of 3001 individuals (mean age 50±10 years, 49% women) from the Framingham Offspring and Third Generation cohorts underwent computed tomography for quantification of periaortic fat and aortic dimensions. We estimated the association between quantitative periaortic and visceral adipose tissue volumes (per standard deviation [SD] increment of volume) with aortic dimensions in both the thorax and abdomen. Thoracic periaortic fat was associated with higher thoracic aortic dimensions (β coefficient per SD of fat volume 0.67 mm, 95% confidence interval 0.58 to 0.76 mm; *P*<0.001). The association persisted after adjustment for age, sex, and cardiovascular risk factors including body mass index and visceral adipose tissue volume. Results for the association of periaortic fat and abdominal aortic dimensions were similar. Further adjustment for adipokines (resistin and adiponectin) had no significant impact on these associations.

**Conclusions:**

Periaortic fat volume was associated with aortic dimensions in both the thorax and abdomen, supporting the notion that local fat depots may contribute to aortic remodeling. Further work to understand the mechanisms underlying this association is warranted.

## Introduction

Diseases of the aorta are a major contributor to cardiovascular mortality, causing nearly 16 000 deaths per year in the United States.^1^ Aortic aneurysms are common, are often clinically silent, and can frequently lead to sudden death.^1^ Although generalized obesity, as measured by body mass index (BMI), has been inconsistently associated with abdominal aortic aneurysms (AAAs),^2–4^ central obesity appears to be a much better marker for aortic aneurysm,^2^ suggesting a potential role for ectopic fat depots in the pathogenesis of AAA.

The mechanism for this association has not been well characterized and may be mediated by local fat depots in proximity to the aorta.^2^ Studies of aortic aneurysms have revealed a prominent inflammatory infiltrate in the adventitia and media, a proinflammatory cytokine profile, increased proteolytic activity, and a resultant loss of tensile strength in the aortic wall.^5,6^ Whereas intraluminal factors such as traditional atherosclerosis risk factors are thought to provide the initiating inflammatory stimulus, emerging evidence suggests that perivascular factors may also play an important role.^7^

Recent studies have shown that periaortic fat, which is in contiguity with the aorta and thus may mediate local effects on aortic remodeling, is associated with both aortic calcification and prevalent peripheral vascular disease.^8,9^ However, the relation between perivascular adipose tissue volume and aortic dimensions, an important predictor for future AAA,^10^ has not been evaluated in a large community-based sample. Accordingly, we evaluated the association between periaortic adipose tissue, using a volumetric imaging protocol, and thoracic and abdominal aortic dimensions in >3000 participants enrolled in the Framingham Heart Study Offspring and Third Generation cohorts.

## Methods

### Study Sample

In 1948, the Framingham Heart Study enrolled 5209 individuals into a longitudinal cohort study. Subsequently, in 1971, the Framingham Offspring study enrolled 5124 children and spouses of the children of the original cohort. In 2002, the Framingham Third Generation study enrolled 4095 children of the Offspring cohort. The study design and entry criteria for each cohort have been described previously.^11–13^ Between June 2002 and April 2005, 3529 Framingham Offspring and Third Generation participants participated in the Multi-Detector Computed Tomography (MDCT) substudy, which has been described previously.^14^ Of these, 3435 participants had periaortic fat quantified. All 3435 had data on aortic dimensions. To be included in the analysis, participants needed to have a complete profile of thoracic periaortic adipose tissue, abdominal periaortic adipose tissue, visceral abdominal adipose tissue, and BMI (ie, no missing values) and then a complete profile of abdominal and thoracic dimensions. Of the 3435, 3006 had complete profile of the fat measurements. Of these, 3004 had no missing thoracic and abdominal dimensions. The total number of subjects in the thoracic analysis was 3001, because 3 of the 3004 subjects were missing risk factors (and for abdominal analysis, 1 subject was further removed because of abdominal surgery, yielding 3000 participants for the abdominal aorta analysis).^11,13^ Excluded participants were marginally older and had a slightly increased prevalence of all cardiac risk factors.

The institutional review boards of Boston University Medical Center and Massachusetts General Hospital approved the study protocol. Written informed consent was provided by all participants.

### Multidetector Computed Tomography Scan Protocol and Measurement of Aortic Dimensions

Chest and abdominal CT imaging was performed using an 8-slice MDCT scanner (LightSpeed Ultra, General Electric, Milwaukee, WI). Using prospective ECG triggering, a noncontrast-enhanced CT scan of the thorax was performed from the level of the carina to the diaphragm and reconstructed in 2.5-mm-thick, nonoverlapping axial images. Similarly, the abdomen was imaged from the upper edge of the S1 vertebrae superiorly using 25 continuous axial slices with a 5.0 mm thickness. In nonobese subjects (<220 lbs), tube voltage 120 kVp and a tube current 320 mA were used.

Measurements of the diameter of the ascending thoracic aorta were acquired at the level of the right pulmonary artery. Measurements of the abdominal aorta were acquired at a level 5 cm above the aortoiliac bifurcation (mid–abdominal aorta). Anteroposterior and transverse measurements were traced from outer edge to outer edge of the aortic wall for the thoracic and abdominal aorta. The mean value of these measurements was used as the summary measure for each aortic dimension. Intra- and interobserver reliability was high for all aortic measurements (0.97 and 0.96, respectively).

### Measurement of Periaortic Fat and Visceral Abdominal Fat Volume

**Figure 1.**
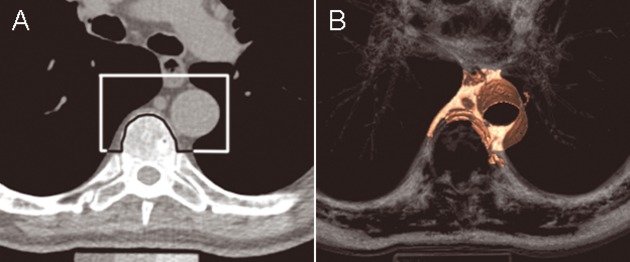
(A) Periaortic fat in an axial CT image (upper boundary) with (B) corresponding 3D reconstruction. Used with permission from Fox et al.^8^

Using a semiautomated method on a dedicated workstation (Aquarius 3D, TeraRecon, San Mateo, CA), image analyses for adipose tissue quantification was performed as previously described.^15^ In brief, CT attenuation thresholds (window width −195 to −45 Hounsfield units) were used to identify adipose tissue. Thoracic periaortic adipose tissue (TAT) borders were manually defined and included the area immediately surrounding the thoracic aorta anteriorly, to the right lateral border of the vertebral body laterally, and to the anterior edge of the vertebral body posteriorly, producing a 6.75-cm column of fat (27 slices) surrounding the thoracic aorta. Abdominal periaortic fat (AAT) was also quantified using similar methods^15^ (see Figure 1). However, because of the close relationship between abdominal periaortic fat and the aortic diameter, we measured this fat depot using concentric rings calibrated to vessel diameter. The inability to resolve the retroperitoneal lining and the inherent correlations between abdominal aortic diameter and quantification of abdominal periaortic fat volume limit the reliability of abdominal fat measures. These measures were therefore considered secondary, and we used thoracic periaortic fat as a proxy for abdominal periaortic fat in primary analyses relating periaortic fat to abdominal aortic dimensions. Reproducibility of periaortic fat volume was excellent for intra- and interreader measurements, with intraclass correlation coefficients of 0.99 and 0.98, respectively.^15^ Visceral abdominal fat (VAT) was quantified as previously described.^16^ Briefly, the abdominal muscular wall was manually traced to separate the subcutaneous from the VAT depot. A semiautomatic quantification of fat volumes with a window width of −195 to −45 Hounsfield units was used. The reproducibility of VAT was excellent, as previously reported.^9^

### Risk Factors

Risk factors were measured at the seventh examination (1998–2001) and the first examination (2002–2005) for members of the Framingham Offspring and Third Generation Cohorts, respectively. BMI was defined as the weight (in kilograms) divided by height (in meters squared). Fasting blood samples were collected for both blood glucose and total cholesterol/high-density lipoprotein ratio. Diabetes was defined as a fasting plasma glucose ≥126 mg/dL or hypoglycemic treatment. Cigarette smoking was categorized as current, former, or never based on a questionnaire administered by a study physician. Current smokers were defined as participants who smoked at least 1 cigarette per day in the year prior to their seventh examination. Former smokers were defined as participants who had smoked in the past but not in the year prior to their seventh examination. Never smokers were defined as individuals who never smoked. Hypertension was defined as systolic blood pressure ≥140 mm Hg, diastolic blood pressure ≥90 mm Hg, or antihypertensive therapy. Circulating fasting plasma levels of resistin and adiponectin were quantified by enzyme-linked immunosorbent assay. Intra-assay coefficients of variation were 9.0% for resistin and 5.8% for adiponectin. Correlations between risk factors and all metabolic traits have previously been reported.^17^

### Statistical Analysis

Periaortic fat (thoracic and abdominal) and visceral abdominal fat were normally distributed and were standardized within each sex to a mean of 0 and a standard deviation of 1 to allow comparisons of the effect estimates between fat depots from regression models. Linear regression models were fitted to assess the association between thoracic TAT, AAT, and VAT volumes (per 1-SD increment of fat volume) and aortic dimensions. Separate models were fitted to model abdominal and thoracic aortic dimensions. The multivariable linear regression model included the covariates of age, sex, smoking (as current, former, or never), diabetes, hypertension, total and high-density lipoprotein cholesterol and lipid treatment. Additional models included BMI or VAT as covariates. We also considered age and sex interactions with periaortic fat for all models described.

In secondary analyses, we examined a subset of individuals with resistin and adiponectin levels (n=965 individuals) to evaluate whether circulating adipokines could explain the associations between fat depots and aortic dimensions. We fitted new linear regression models, adding log-transformed resistin and adiponectin levels to the other covariates (as outline above), and evaluated the impact of these adipokines on the effect estimates for the associations between fat depots and aortic dimensions. All analyses were performed with SAS version 9.1.3. *P*<0.05 was considered statistically significant.

## Results

**Figure 2.**
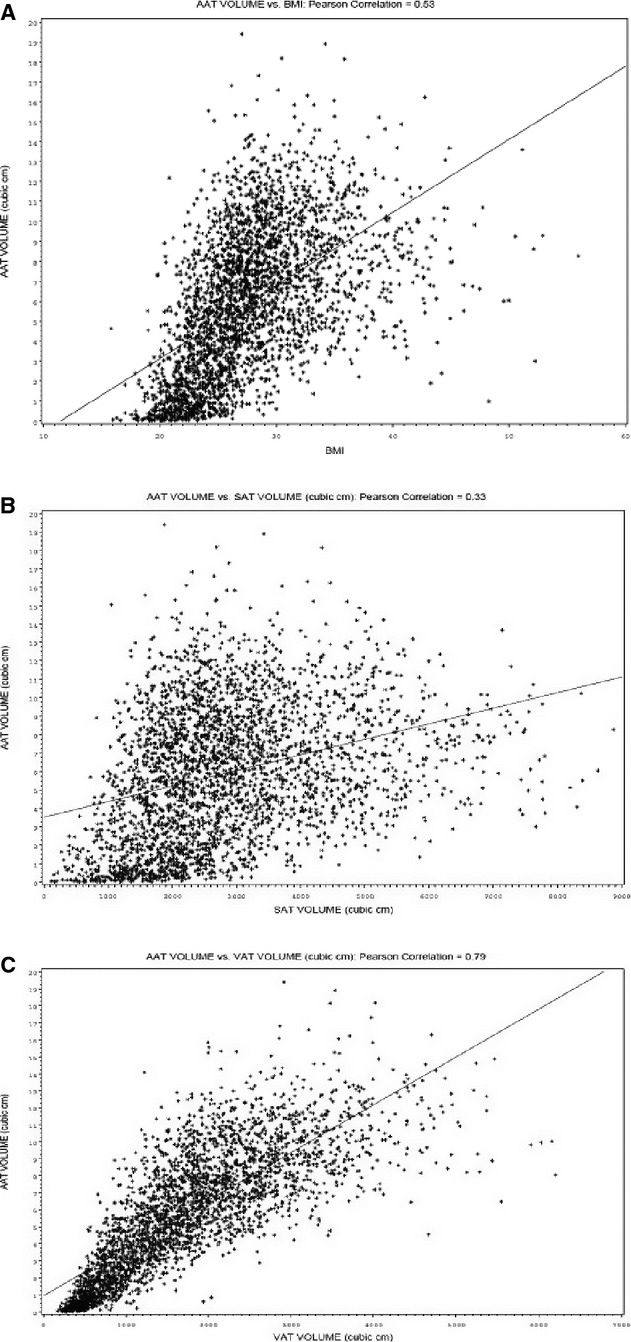
Correlations between (A) AAT and BMI, (B) AAT and SAT, and (C) AAT and SAT. AAT indicates abdominal periaortic fat; BMI, body mass index; and SAT, subcutaneous adipose fat area.

The study comprised 1474 women and 1527 men with a mean age of 52±10 years and 49±10 years, respectively. Mean ascending thoracic aortic dimensions was 23±2 mm in women and 26±3 mm in men. Mean abdominal aortic dimensions was 17±2 mm in women and 19±2 mm in men. All characteristics of the sample are summarized in Table 1. We identified significant correlations between all fat indices, including local and global measures of adipose tissue, with thoracic aortic dimensions (Table 2, Figure 2). On average, the correlation between adiposity measures and aortic diameter was stronger in the thoracic aorta than in the abdominal aorta.

**Table 1.** Clinical Characteristics of Overall Sample

	Women	Men
N	1474	1527

Age, years	52±10	49±10

Body mass index, kg/m^2^	27±6	28±4

Smoking (current/former/never), n (%)		

Current	176 (12)	198 (13)

Former	631 (43)	535 (35)

Never	667 (45)	794 (52)

Systolic blood pressure, mm Hg	120±18	123±14

Total cholesterol/HDL, mg/dL	3.4±1.1	4.5±1.4

Diabetes, n (%)	81 (7)	81 (5)

Hypertension treatment, n (%)	270 (18)	273 (18)

Lipid-lowering treatment, n (%)	146 (10)	252 (17)

History of myocardial infarct, n (%)	8 (0.5)	40 (3)

Aorta dimension, mm		

Thoracic	23±2	26±3

Abdominal	17±2	19±2

Fat volumes, cm^3^		

TAT	9.8±5.2	16.9±8.3

AAT	4.0±2.9	7.9±3.2

VAT	1350±828	2197±996

Values represent means±standard deviations except where otherwise specified. TAT indicates thoracic periaortic adipose tissue; AAT, abdominal periaortic fat; and VAT, visceral abdominal fat.

**Table 2.** Age- and Sex-Adjusted Correlations Between Indices of Adiposity and Aortic Dimensions

	Aortic Dimensions	
	
	Thoracic	Abdominal
TAT	0.26	0.14

AAT	0.23	0.10

VAT	0.28	0.13

BMI	0.34	0.22

WC	0.33	0.21

*P*<0.001 for all comparisons.

TAT indicates thoracic periaortic adipose tissue; AAT, abdominal periaortic adipose tissue volume; VAT, visceral adipose tissue volume; BMI, body mass index; WC, waist circumference.

### Associations of Regional Adipose Tissue Volumes With Thoracic Aortic Dimensions

In age- and sex-adjusted models, TAT and VAT were associated with thoracic aortic dimensions (P<0.001) (Table 3). We found that for each standard deviation increment in thoracic periaortic fat, there was an associated increase of 0.67 mm (95% confidence interval [CI] 0.58 to 0.76 mm) in thoracic aortic dimensions. These associations persisted after adjustment for cardiometabolic risk factors and BMI. When VAT was added in the same model with TAT, TAT remained significantly associated with thoracic aortic dimensions (β coefficient 0.24, 95% CI 0.11 to 0.37; *P*<0.001 for TAT), whereas VAT did not (β coefficient 0.04, 95% CI −0.11 to 0.19; *P*=0.59). No significant interactions by age and sex were identified (all interaction *P*>0.05 by age and sex in fully adjusted models). For thoracic aortic dimensions, age, sex, cardiovascular risk factors, and BMI explained 56% of the variance. The addition of TAT to these covariates increased the *R*^2^ by 0.4%.

**Table 3.** Association of Adipose Tissue Volumes With Thoracic Aortic Dimensions

	Age- and Sex Adjusted		Risk Factor Adjusted*		+BMI Adjusted		+Fat Deposit Adjusted	
				
	β (95% CI)	*P*	β (95% CI)	*P*	β (95% CI)	*P*	β (95% CI)	*P*
TAT	0.67 (0.58 to 0.76)	<0.001	0.59 (0.50 to 0.69)	<0.001	0.26 (0.16 to 0.37)	<0.001	0.24 (0.11 to 0.37)†	<0.001

VAT	0.68 (0.60 to 0.77)	<0.001	0.65 (0.56 to 0.74)	<0.001	0.21 (0.09 to 0.33)	<0.001	0.04 (−0.11 to 0.19)‡	0.59

TAT indicates thoracic periaortic adipose tissue volume; VAT, visceral adipose tissue volume; and BMI, body mass index.

* Adjusted for the following covariates: age, sex, smoking (current, former, or never), systolic blood pressure, blood pressure treatment, total cholesterol/HDL ratio, lipid treatment, diabetes).

† Fat-deposit-adjusted model included VAT and was adjusted for all other covariates (including BMI).

‡ Adjusted for TAT and all other covariates (including BMI). All β coefficients presented are per 1 standard deviation change in fat volume.

### Associations of Regional Adipose Tissue Volumes and Abdominal Aortic Dimensions

In age- and sex-adjusted models, TAT and VAT were associated with abdominal aortic dimensions (*P*<0.001 for all) (Table 4). These associations persisted after adjustment for cardiovascular risk factors. However, after adjustment for BMI, only TAT remained significantly associated with abdominal aortic dimensions (β coefficient 0.09, 95% CI 0.01 to 0.18; *P*=0.04). Furthermore, when VAT was added to the models with TAT, TAT remained associated with abdominal aortic dimensions (β coefficient 0.19, 95% CI 0.08 to 0.30; *P*<0.001), whereas VAT became negatively associated (β coefficient −0.18, 95% CI −0.31 to −0.06; *P*=0.004). These results were consistent in both men and women and in younger (<50 years) and older (≥50 years) individuals (interaction *P*>0.05 for both).

**Table 4.** Association of Adipose Tissue Volumes With Abdominal Aortic Dimensions

	Age- and Sex Adjusted		Risk Factor Adjusted*		+BMI Adjusted		+Fat Deposit Adjusted	
				
	β (95% CI)	*P*	β (95% CI)	*P*	β (95% CI)	*P*	β (95% CI)	*P*
TAT	0.29 (0.22 to 0.37)	<0.001	0.31 (0.23 to 0.39)	<0.001	0.09 (0.01 to 0.18)	0.04	0.19 (0.08 to 0.30)†	<0.001

VAT	0.25 (0.19 to 0.32)	<0.001	0.29 (0.22 to 0.37)	<0.001	−0.05 (−0.15 to 0.05)	0.30	−0.18 (−0.31 to −0.06)‡	0.004

*Secondary Analysis*								

AAT	0.22 (0.14 to 0.29)	<0.001	0.26 (0.18 to 0.33)	<0.001	0.02 (−0.07 to 0.10)	0.71	0.05 (−0.05 to 0.16)†	0.29

TAT indicates thoracic periaortic adipose tissue volume; VAT, visceral adipose tissue volume; and AAT, abdominal periaortic adipose tissue volume.

* Adjusted for the following covariates: age, sex, smoking (current, former, or never), systolic blood pressure, blood pressure treatment, total cholesterol/HDL ratio, lipid treatment, diabetes).

† Fat-deposit-adjusted model included VAT and was adjusted for all other covariates (including BMI).

‡ Adjusted for TAT and all other covariates (including BMI). All β coefficients presented are per 1 standard deviation change in fat volume.

As a secondary analysis, we also evaluated our measure of abdominal periaortic fat with abdominal aortic dimensions. The relations between AAT and abdominal aortic dimensions were largely the same as those reported for TAT and abdominal aortic dimensions (Table 4). In addition, we also examined AAT and VAT categories as defined by the median. Among both high and low VAT, mean abdominal dimensions were larger in the high AAT category (*P*<0.001 for all comparisons). For abdominal aortic dimensions, age, sex, risk factors, and BMI explained 46.8%; with the addition of periaortic fat, this was increased by 0.1%.

### Circulating Resistin and Adiponectin in Associations Between Regional Fat Deposits and Aortic Dimensions

In secondary analyses, to determine whether circulating adipokines could explain the association between fat depots and aortic dimensions, we examined these associations in participants with adipokines available (n=965). In this sample, the associations between TAT and aortic dimensions were largely the same as for the entire sample. Adjustment for resistin and adiponectin levels did not materially change the reported associations (Tables 5 and 6).

**Table 5.** Association of Adipose Tissue Volumes With Thoracic Aortic Dimensions in Subset of Individuals With Adipokines

	Age- and Sex- and RF Adjusted*		+BMI†		+Adipokines†	
			
	β (95% CI)	*P*	β (95% CI)	*P*	β (95% CI)	*P*
TAT	0.56 (0.38 to 0.74)	<0.001	0.24 (0.05 to 0.44)	0.02	0.24 (0.05 to 0.44)	0.02

VAT	0.61 (0.44 to 0.78)	<0.001	0.23 (0.02 to 0.44)	0.04	0.23 (0.02 to 0.44)	0.04

TAT indicates thoracic periaortic adipose tissue volume; VAT, visceral adipose tissue volume; and BMI, body mass index.

* Adjusted for the following covariates: age, sex, smoking (current, former, or never), systolic blood pressure, blood pressure treatment, total cholesterol/HDL ratio, lipid treatment, diabetes). All β coefficients presented are per 1 standard deviation change in fat volume.

† For +BMI and +adipokines, BMI and adipokines were added serially in nested models (first BMI was added to the baseline model, then log [adiponectin] and log [resistin] were added).

**Table 6.** Association of Adipose Tissue Volumes With Abdominal Aortic Dimensions in Subset of Individuals with Adipokines

	Age- and Sex- and RF Adjusted*		+BMI†		+Adipokines†	
			
	β (95% CI)	*P*	β (95% CI)	*P*	β (95% CI)	*P*
TAT	0.33 (0.18 to 0.47)	<0.001	0.12 (−0.04 to 0.28)	0.15	0.13 (−0.03 to 0.29)	0.11

VAT	0.27 (0.13 to 0.40)	<0.001	−0.05 (0.22 to 0.12)	0.56	−0.03 (−0.20 to 0.14)	0.71

*Secondary Analysis*						

AAT	0.25 (0.11 to 0.39)	<0.001	0.06 (−0.09 to 0.21)	0.45	0.07 (−0.08 to 0.22)	0.39

BMI indicates body mass index; TAT, thoracic periaortic adipose tissue volume; VAT, visceral adipose tissue volume; and AAT, abdominal periaortic adipose tissue volume.

* Adjusted for the following covariates: age, sex, smoking (current, former or never), systolic blood pressure, blood pressure treatment, total cholesterol/HDL ratio, lipid treatment, diabetes). All β coefficients presented are per 1 standard deviation change in fat volume.

† For +BMI and +adipokines, BMI and adipokines were added serially in nested models (first BMI was added to the baseline model, then log [adiponectin] and log [resistin] were added).

## Discussion

In this community-based sample of >3000 individuals, periaortic fat depots in the thorax and abdomen were associated with aortic dimensions. These novel associations persisted after adjustment for age, sex, and cardiovascular risk factors. In addition, although generalized and central obesity are risk factors for aortic aneurysm,^2–4^ we found that periaortic fat remained associated with aortic dimensions even after adjustment for BMI, a measure of generalized obesity, and VAT, a measure of central obesity. Our results suggest that local fat depots contiguous to the aorta may have local effects on aortic remodeling over and above the systemic effects of generalized and/or central obesity. In addition, we determined that the associations between periaortic fat and aortic dimensions do not appear to be mediated via circulating levels of adipokines, further supporting a possible local, as opposed to systemic, effect of adipose tissue.

Because of the limitations in abdominal aortic fat quantification and the inherent correlation between abdominal aortic diameter and periaortic fat, we used thoracic periaortic fat, which is not affected by these technical limitations in quantification, as a proxy for periabdominal fat and identified associations with abdominal aortic dimensions. It is reassuring that in secondary analyses where we used abdominal periaortic fat, similar associations were found, suggesting that the use of thoracic periaortic fat may be a reasonable proxy for abdominal periaortic fat. Alternative methods to accurately quantify abdominal periaortic fat without these limitations warrant further study.

### In the Context of the Current Literature

Obesity, as measured by BMI, has been associated with aortic aneurysms and increased aortic dimensions in previous reports.^2^ However, this association has been inconsistent across studies.^3,4^ Golledge et al reported that BMI is a significant correlate of aortic dimensions in univariate analyses,^2^ and Lederle et al reported that BMI is a weak correlate for AAA after multivariate adjustment for other risk factors.^4^ Moreover, in a prospective cohort study of >100,000 individuals with >600 AAAs, Irribaren et al found no association between BMI and incident AAA.^3^ Although generalized measures of obesity such as BMI appear to be poor predictors of aortic disease, central obesity, as measured by waist-to-hip ratio or waist circumference, appears to be a better predictor for AAA. Waist-to-hip ratio and waist circumference have been shown to increase the odds ratio for the presence of an enlarged aorta (defined as >30 mm) by 14% and 22%, respectively.^2^ Our results extend these findings by showing that ectopic adipose tissue quantified by computed tomography and, specifically, periaortic adipose tissue is associated with aortic dimensions even after adjustment for other vascular and metabolic risk factors including global measures of obesity such as BMI. Although we used aortic dimensions and not clinically defined aneurysms as our outcome, aortic dimensions have been shown to predict future aortic aneurysm repair or death.^10^

Our findings add to the growing body of literature in support of a local effect of adipose tissue. Previous studies have shown that pericardial fat is associated with coronary artery calcification,^18^ left atrial dimensions,^19^ and prevalent atrial fibrillation.^20^ In addition, we have recently reported that periaortic fat, but not visceral abdominal fat, is associated with aortic calcification and peripheral vascular disease after adjustment for cardiometabolic risk factors.^8,9^ We now extend these findings and demonstrate a novel relation between periaortic fat and aortic dimensions.

We found that the association between periaortic fat and aortic dimensions was not likely mediated via circulating systemic levels of adiponectin or resistin, which have been shown to be associated with AAA.^2,21^ We cannot rule out that resistin and other mediators secreted locally by adipocytes and activated macrophages may promote local effects on the aorta that are not measurable in the systemic circulation. Efforts to understand the effect of local mediators via molecular imaging techniques^22^ and other methods warrant further investigation.

### Potential Mechanisms

Inflammation represents a major pathophysiologic mechanism in aortic wall remodeling and aortic dilatation.^23^ Periaortic adipose tissue, which surrounds the aorta, is characterized by reduced differentiation of adipocytes, increased inflammatory cytokine production, and downregulated anti-inflammatory adipokines.^24^ In addition, perivascular adipose tissue surrounding human atherosclerotic aortas has been shown to have a significantly greater inflammatory cell infiltrate compared with normal aorta and may represent a source for the adventitial inflammatory infiltrates seen in AAA.^25^ Animal studies have shown that vascular adventitia also contain metabolically active adipoctyes that originate from periaortic adipose tissue and could modulate local vascular remodeling.^24^ Periaortic adipose tissue may therefore represent a local source of proinflammatory cells and mediators. The resultant adventitial inflammation could promote aortic wall degeneration and aortic dilatation. In keeping with these previous reports, recent studies have shown that obesity induced by high-fat feeding in mice led to markedly increased macrophage infiltration and cytokine expression in periaortic fat with increased development of aortic aneurysms.^24^ These findings suggest that the effects of obesity may be mediated via local changes in periaortic fat, which modulate the balance of pro- and anti-inflammatory signals and ultimately lead to the aortic remodeling that characterizes aortic aneurysms.

### Strengths and Limitations

Strengths of the present analysis consist of detailed characterization of regional fat deposits by CT in a large community-based sample with detailed risk factor data available. However, several limitations deserve mention. First, our measure of abdominal periaortic fat was not as reliable as thoracic periaortic fat because of technical limitations in quantification. For analyses in which periaortic fat was related to abdominal aortic dimensions, we used thoracic periaortic fat as a proxy for abdominal periaortic fat. The consistency of our results using either thoracic or abdominal periaortic fat suggests that this is a reasonable approach. Second, we were unable to relate periaortic fat to clinical aortic aneursyms in this middle-aged community-based sample of ambulatory individuals because of the very low prevalence in our sample. However, we used aortic dimension as an outcome, an important predictor for future AAAs.^10^ Third, our sample was predominantly of white European descent and therefore results may not apply to other ethnicities or racial groups. Fourth, only a subset of individuals had adipokines available for analysis, limiting statistical power in these analyses. Nonetheless, our findings in this smaller sample were consistent with the main findings and were not materially changed by the addition of adipokines to the multivariable models. In addition, other important biomarkers such as metalloproteinases or novel functional imaging modalities such as positron emission scanning were not available to further explore possible mechanisms responsible for our reported association. Fifth, because of the relatively high correlations between different measures of adiposity, collinearity during modeling may have limited the interpretations of some of our results. For example, we noted that when VAT and TAT were added in the same model with abdominal aortic dimensions, both remained significant, but VAT became negatively correlated with abdominal dimensions. It is highly unlikely that VAT is truly associated with smaller aortic dimensions after adjusting for TAT. Although we found that periaortic fat is associated with aortic dimensions after adjustment for BMI and other fat depots, it is possible that the inclusion of several ectopic fat depots (eg, periaortic fat and VAT) may better predict aortic dimensions than either fat depot alone. Finally, our study was cross-sectional and observational; therefore, although periaortic fat was associated with aortic dimensions, causation cannot be inferred.

## Conclusions

In this community-based sample of >3000 individuals with volumetric CT quantification of periaortic and visceral fat, periaortic fat volume was associated with both thoracic and abdominal aortic dimensions. This association persisted after adjustment for cardiovascular risk factors, BMI, and visceral adipose tissue volume, indicating that periaortic fat volume may be a novel correlate for aortic dilatation. Our findings are consistent with the hypothesis that periaortic adipose tissue may influence aortic remodeling via local mechanisms. Further research to identify the local mediators and to further understand the local mechanisms mediating this association is warranted.
